# Is Epithelial-Mesenchymal Transition a New Roadway in the Pathogenesis of Oral Submucous Fibrosis: A Comprehensive Review

**DOI:** 10.7759/cureus.29636

**Published:** 2022-09-26

**Authors:** Abikshyeet Panda, Pallavi Mishra, Aishwariya Mohanty, Krishna Sireesha Sundaragiri, Arpita Singh, Kunal Jha

**Affiliations:** 1 Department of Oral Pathology, Kalinga Institute of Dental Sciences, Kalinga Institute of Industrial Technology (KIIT) Deemed to be University, Bhubaneswar, IND; 2 Department of Oral Pathology, Rajasthan University of Health Sciences College of Dental Sciences, Jaipur, IND; 3 Department of Public Health Dentistry, Kalinga Institute of Dental Sciences, Kalinga Institute of Industrial Technology (KIIT) Deemed to be University, Bhubaneswar, IND

**Keywords:** oral squamous cell carcinoma, therapeutic target, pathogenesis, oral submucous fibrosis (osmf/osf), epithelial-mesenchymal transition (emt)

## Abstract

Epithelial-mesenchymal transition (EMT) collectively refers to a series of episodes that reshape polarized, intact epithelial cells into discrete motile cells that can conquer the extracellular matrix (ECM). It performs a pivotal role in embryonic development, wound healing, and tissue repair. Surprisingly, the exact mechanism can also lead to the onset of malignancy and organ fibrosis contributing to scar formation and loss of function. transforming growth factor signaling, WNT signaling, Notch signaling, Hedgehog signaling, and receptor tyrosine kinase signaling, as well as non-transcriptional changes in response to extracellular cues, such as growth factors and cytokines, hypoxia, and contact with the surrounding ECM, are responsible for the initiation of EMT.

Although the pathogenesis of oral submucous fibrosis (OSMF) is multifactorial, compelling evidence suggests that it results from collagen deregulation. EMT is one of the spotlight events in the pathogenesis of OSMF, with myofibroblasts and keratinocytes being the victim cells. EMT is an essential step in both physiological and pathological events. The importance of EMT in the malignant development of OSMF and the inflammatory reaction preceding fibrosis implies a new upcoming area of research.

This review aims to focus on the EMT events that function as a double-edged sword between wound healing and fibrosis and further discuss the mechanisms along with the molecular pathways that direct changes in gene expression essential for the same in the oral cavity. As OSMF involves a risk of malignant transformation, understanding the cellular and molecular events will open more avenues for therapeutic breakthroughs targeting EMT.

## Introduction and background

“Cells come from cells.” This 145-year-old concept is so fundamental that it is implicitly accepted even today by biologists. Normally cells divide asymmetrically and develop along destined routes, or they may undergo oncogenesis after the establishment of normal tissues. Epithelial-mesenchymal transition (EMT) is a distinct cellular entity that permits disaggregation of epithelial units and reshaping of epithelia for movement, i.e., acquisition of mesenchymal traits [1]. Among the three types of EMT, each with its own set of functional consequences, type II represents wound healing as part of the repair-related event, which generates fibroblasts and other associated cell lines to regenerate tissues in response to epithelial stress, trauma, or inflammatory insult. EMT is not only a physiological response to injury but is also a significant pathological event involving organ degeneration such as fibrosis [2,3]. Fibrosis is the result of chronic inflammatory reactions induced by various stimuli, including persistent infections, autoimmune reactions, allergic responses, chemical insults, radiation, and tissue injury [4]. The drawback of excess deposition of collagen fibers is that the epithelial units are surpassed by scarification and lose their morphology and identity, leaving involved organs and tissue to fail [5]. Although all three types of EMT have the same molecular basis, type I EMT is a physiological event that occurs during organogenesis and then goes through a mesenchymal-epithelial transition (MET) to generate secondary epithelia, resulting in EMT reversal and gene expression. Upregulation of extracellular matrix (ECM) proteins and transcription factors characterize type II EMT, resulting in a phenotypic transition of epithelial cells that ultimately leads to fibrosis. Type III EMT undergoes phenotypic changes because of the activation of EMT-related genes, which increase cancer cell tissue invasion and metastasis [6,7]. Oral submucous fibrosis (OSMF) is a prolonged, serious, debilitating, potentially malignant condition primarily affecting Indians, possibly because of chronic use of areca nut, resulting in significant rigidity and difficulty to open the mouth. Its pathogenesis is believed to be multifactorial. It illustrates a wound-healing process in response to sustained chronic injury which is represented by generalized fibrosis due to the paradigm shift of the secreted inflammatory mediators that initiates EMT [7,8].

The pathogenesis of OSMF is multifactorial with EMT being one of the major events that result in collagen deregulation due to abnormal changes in myofibroblasts and keratinocytes. Removal of the causative agent causing chronic injury along with various therapies is the chief treatment modality. This review presents the cross-talk between tissue repair and fibrosis as well as reviews the molecular mechanisms involved, along with the potential role of EMT in clinical application with a focus on OSMF.

## Review

An overview of epithelial-mesenchymal transition

**Figure 1:**
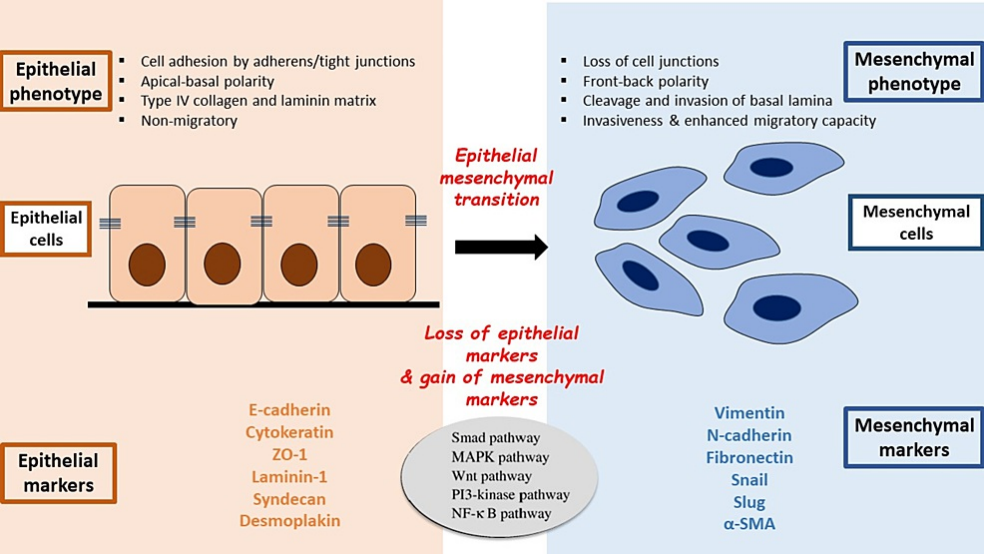
Cellular and molecular changes associated with epithelial-mesenchymal transition. Image credits: Aishwariya Mohanty.

EMT can be defined as “A biological process that allows a polarized epithelial cell, which normally interacts with the basement membrane via its basal surface, to undergo multiple biological changes that enable it to resume a mesenchymal cell phenotype” [2]. Once epithelial cells are capable of reacting to EMT-instigating signals, these steps can cause the disorganization of the intercellular adhesion complexes which ultimately leads to the loss of their distinct apicobasal polarity [9] (Figure 1). Large alterations at the cellular level, such as activation of certain transcription factors, microRNA production, epigenetic modifications, and post-translational modification protein, are essential to bringing about EMT [10,11]. Microenvironmental cues and inducing signals that interact with epigenetic regulators are also required for the activation of EMT. The function of epigenetic regulators is to modify the protein expression involved in diverse processes (such as cell polarity, cell-cell adhesion, cell-cell contact, cytoskeleton, and ECM degradation), as well as to suppress the expression of key epithelial markers to give them migratory and invasive properties [12]. The EMT process is reversible and is known as MET, which occurs both during the development of the embryo as well as during disease pathophysiology. This reversible process highlights the adaptability of certain embryonic cells as well as adult cells that help in disease pathogenesis as well as a therapeutic intervention [13].

Biomarkers for epithelial-mesenchymal transition

Cell Surface Markers

The most common epithelial marker for EMT is a change in E-cadherin expression. Its function is reduced during EMT in fetal development, tissue fibrosis, and neoplastic conditions. Furthermore, its absence has been shown to promote EMT [5]. The switch from E-cadherin to N-cadherin, which is expressed in mesenchymal cells, fibroblasts, cancer cells, and neural tissue, has been used as an EMT marker. EMT has also been linked to cell relocation from the basement membrane microenvironment into the fibrillary ECM, as evidenced by changes in the levels of expression of various integrins (integrin switch). Furthermore, integrin signaling promotes EMT but is expressed by both epithelial and mesenchymal cells. As a result, they have limited utility as EMT markers but can be used in a context-dependent manner, for example, in colon cancer, only carcinoma cells that have undergone type III EMT express high levels of β6-integrin, whereas normal epithelial cells and non-invasive cancer cells express moderate levels [8].

Discoidin domain receptor 2 (DDR2), a collagen-specific receptor tyrosine kinase (RTK), is a marker of adaptation to the altered ECM environment associated with EMT. Its expression regulates matrix metalloproteinase-1 (MMP-1) and cell motility, and it correlates with increased cancer cell invasiveness [7].

Cytoskeletal Markers

Fibroblast-specific protein-1 (FSP-1) is a member of the calcium-binding S-100 protein family and is a marker for the detection of EMT in cancer and fibrosis. In tissue fibrosis, FSP-1 expression is demonstrable early in the transition to fibroblast, as reported in kidney and liver fibrosis. In cancer, ectopic expression of FSP-1 facilitates type III EMT in cancer cells [7]. Vimentin is an intermediate filament protein that is found in various cells, including fibroblasts, endothelial cells, hematopoietic cells, and glial cells. The positive expression of vimentin is used to identify epithelial cells undergoing type III EMT in cancer [5].

During embryonic development, the de-novo expression of alpha-smooth muscle actin (α-SMA) has been demonstrated in EMT that inevitably leads to the cardiac cushion. Type II EMT is also linked to myofibroblasts that express α-SMA in tissue fibrosis. Evidence for α-SMA exists in breast cancers, where this molecule has been detected primarily in basal phenotype breast tumors [5].

β-catenin is a cytoplasmic plaque protein that connects cadherins to the cytoskeleton. It also acts as a co-transcriptional activator, playing a dual role in EMT. Its process is governed by mechanisms that control the amount of β-catenin in the cytoplasm, either through recruitment to cadherin-binding partners or degradation. It is normally found in the cytoplasm of normal epithelial cells and non-invasive tumor cells, but in cells undergoing EMT, it is found either in the cytoplasm, indicating E-cadherin dissociation, or in the nucleus, indicating its role as a transcriptional activator. β-catenin has been used as an EMT marker in studies of embryonic development, cancer, and tissue fibrosis because it directly controls the expression of EMT-related genes, particularly Snail 1 (Drosophila homolog, zinc finger protein) [9].

Extracellular Matrix Proteins

Fibronectin is a glycoprotein with a high molecular weight that acts as a substrate for fibrillar ECM. It has been used to detect type I EMT, which is associated with gastrulation, palate fusion, and neural crest migration. Furthermore, both type II and type III EMT are linked to increased fibronectin expression [12].

The basement membrane constituents such as type IV collagen, laminin, and nidogen are downregulated during EMT, suggestive of the invasive and migratory capacity of cells undergoing EMT [9]. Laminin, especially laminin-1, is shown to be downregulated during gastrulation and palate fusion as well as in fibrotic diseases. Laminin-5 upregulation and its expression in a discontinuous pattern have been associated with invasive cancers, and its expression is linked with type III EMT in breast cancer, hepatocellular carcinoma, and oral squamous cell carcinoma (OSCC) [9].

Transcription Factors

Fibroblast transcription site-1 (FTS-1) is a cis-acting regulatory element found in EMT-related genes that encode key players such as FSP-1, twist, Snail 1, E-cadherin, E-catenin, vimentin, α-SMA, and others. In type II and III EMT related to fibrosis and metastatic tumor formation, de novo expression of the core-binding factor-α subunit (CBF-A), a member of the heterogeneous nuclear ribonucleoprotein-A family, can be seen [12].

Snail transcription factors are an example of a common downstream target of various signaling pathways that control EMT. Snail activation appears to be involved in all known EMT events during development, fibrosis, and cancer. Snail inhibits E-cadherin expression while increasing the expression of mesenchymal markers, such as vimentin and fibronectin. Furthermore, they promote basement membrane disruption and invasion by increasing MMP-1 expression and inhibiting apoptosis. The Twist is a basic helix-loop-helix protein that is transcriptionally active during cell differentiation and lineage determination. In all three types of EMT, it is upregulated. Twist suppresses E-cadherin expression while increasing fibronectin and N-cadherin expression during the development of metastatic cancer cells as part of type III EMT [12].

Forkhead box C2 (FOXC2) is another transcription factor that induces EMT. During embryogenesis, FOXC2 expression is required for angiogenesis, musculogenesis, and organogenesis of the kidney, heart, and urinary tract. Upregulation of any of the EMT inducers, i.e., transforming growth factor-beta (TGF-β), Snail, or Twist increases FOXC2 expression, leading to the induction of type III EMT. Its role in fibrosis is not well established [11].

Molecular mechanism and signaling pathways

**Figure 2:**
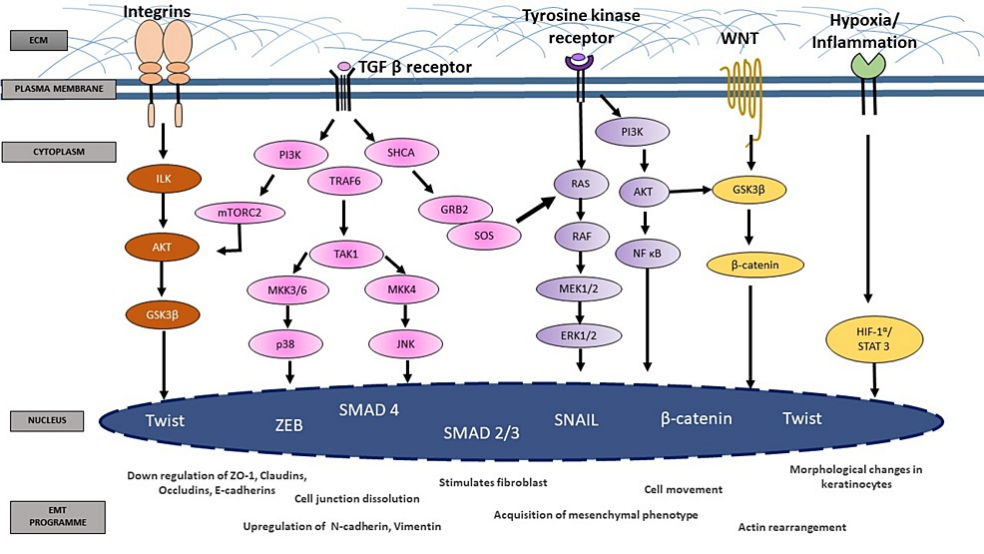
Signaling pathways regulating epithelial-mesenchymal transition mechanism. Image credits: Aishwariya Mohanty.

The epithelial cells can downregulate their epithelial properties and adopt mesenchymal ones [2]. This transformation in cell differentiation and behavior is mediated by transcription factors such as Snail, zinc-finger E-box-binding (ZEB), and basic helix-loop-helix transcription factors, whose actions are carefully regulated at the transcriptional, translational, and post-translational stages. TGF signaling, WNT signaling, Notch signaling, Hedgehog signaling, and RTK signaling, as well as non-transcriptional changes in response to extracellular cues, such as growth factors and cytokines, hypoxia, and contact with the surrounding ECM, initiate and control gene expression reprogramming during EMT [10,12] (Figure 2). They phosphorylate a number of intracellular kinase cascades to trigger transcription factors that activate EMT-associated genes once activated. The TGF family has the greatest impact on wound healing and fibrosis; nevertheless, signaling pathways overlap often. Each TGF subfamily has its own set of receptors as well as a diverse set of Smad signaling proteins [14].

The phosphatidylinositol-3-kinase and protein kinase B (PI3K-AKT), extracellular signal-regulated kinases mitogen-activated protein kinases (ERK-MAPK), p38, and JUN N-terminal kinase (JNK) pathways are all activated by TGF-β [15]. TGF-β R1 phosphorylates the adaptor protein SRC homology 2 domain-containing-transforming A (SHCA), which generates a docking site for growth factor receptor-bound protein 2 (GRB2) and son of sevenless (SOS) and activates the RAS-RAF-MEK-ERK-MAPK cascade. TGF activates p38 and JNK as a result of TNF receptor-associated factor 6 (TRAF6) interacting with the TGF receptor complex, which activates TGF-activated kinase 1 (TAK1) [16]. Postnatally, in the oral cavity, TGF-β1 mediates EMT in wound healing, OSMF, and cancer metastasis [17]. Various studies have shown that EMT signature markers are precisely positive in OSMF cases [18]. Exposure of the oral mucosa to areca nut extract (ANE) chewing may initiate EMT signatures long before clinical manifestations irrespective of the duration or frequency of the habit [8]. According to an in-vitro study, EMT of primary epithelial cells depends on TGF-β expression. The important role of receptor-regulated Smads in TGF-dependent EMT associated with epithelial damage was confirmed in this study. Many studies have also shown that targeting the PI3K pathway prevents TGF-β‑induced EMT, demonstrating the essential role of the PI3K-AKT pathway in this process [19].

Epidermal growth factor (EGF), fibroblast growth factor (FGF), hepatocyte growth factor (HGF), and vascular endothelial growth factor (VEGF) all operate through RTKs to induce EMT. The RAS-RAF-MEK-ERK-MAPK signaling pathway is one of the most important pathways that is activated by RTKs in response to growth stimuli. ERK1 and ERK2-MAPK once activated can promote EMT by boosting the production of EMT transcription factors that regulates cell motility and invasion [20]. WNT signaling promotes EMT by inhibiting glycogen synthase kinase-3 (GSK3), which stabilizes catenin, translocates it to the nucleus, and engages the transcription factors lymphoid enhancer-binding factor 1 (LEF) and T-cell factor (TCF) to promote gene expression program. WNT promotes EMT by increasing β‑catenin-mediated gene expression at the invasive front of tumors [21]. Hedgehog signaling also participates in EMT. Hedgehog and Notch signaling promote a decrease in E-cadherin levels by activating glioma 1 (GLI1), which can induce SNAIL1 (Snail family transcriptional repressor 1) expression, and the intracellular domain of Notch can activate SNAIL2 expression; thus, Hedgehog and Notch signaling promote a decrease in E-cadherin levels [12]. EMT is also influenced by the cell microenvironment. Interleukin-6 (IL-6) can promote EMT by inducing SNAIL1 expression via the Janus kinase (JAK) signal transducer and activator of the transcription 3 (STAT3) pathway during inflammation and malignancy [22]. Hypoxia in the tumor environment can promote EMT by activating Twist expression through hypoxia-inducible factor 1 (HIF1) [10].

Hence, these findings aid in our understanding of the governing networks (both gene and protein levels) involved in EMT during embryonic development and wound healing, which is critical in understanding the regulatory networks involved in fibrosis. However, because TGF-β has pleiotropic biological actions that are mediated by multiple signaling pathways, therapies that target TGF-β expression/activation or TGF-β binding to its receptor may potentially cause numerous unwanted side effects due to the overlapping of various signaling pathways.

Epithelial-mesenchymal transition and wound healing

EMT is a naturally occurring transdifferentiation program that plays a pivotal role in wound healing by secreting inflammatory molecules by the inflammatory cells and fibroblasts. These molecules can transact with collagens, laminins, and elastin and bring about EMT [23]. Inflammatory, proliferative, and maturation phases are involved in the repair and regeneration of the tissue. The inflammatory phase focuses to restrict tissue damage through phagocytosis. The second phase aims at the inception of granulation tissue, angiogenesis, and deposition of new ECM. The key step of wound healing is re-epithelialization which is maintained by the conversion of cells from a sedentary state to the migratory one due to EMT. Normally, the keratinocytes present in the epithelial layer undergo differentiation till it reaches the outer layer. In wound healing, these cells encounter various inflammatory mediators and undergo modification in cellular phenotype-inducing EMT. This is invariably different from that occurring in any tumor. The growth factors that initiates wound healing or primarily EMT include HGF, EGF, insulin-like growth factor, connective tissue growth factor, TNF-α, and FGF [24,25].

TGF-β, one of the major cytokine inducing EMT, has been identified in granulation tissue of wound healing in thermal burns, and it was found that there was high expression of TGF-β receptors in fibroblasts involved in wound repair. This upregulation if surpasses its limit can lead to hypertrophic scars [2].

Epithelial-mesenchymal transition and fibrosis

**Figure 3:**
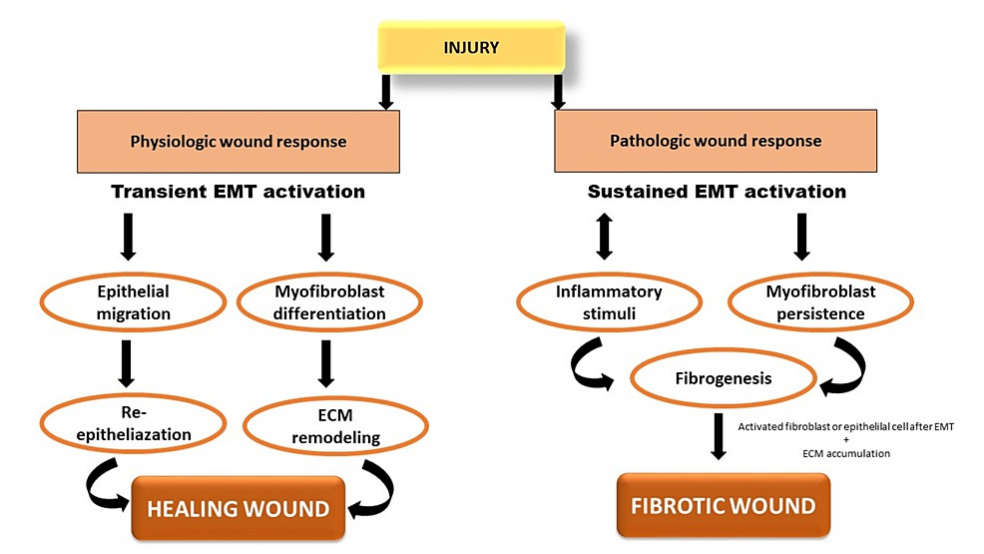
Epithelial-mesenchymal transition process during wound healing and fibrosis. Image credits: Aishwariya Mohanty. EMT: epithelial-mesenchymal transition; ECM: extracellular matrix

Fibrosis is the end result of chronic inflammation and is defined by the overgrowth, hardening, and/or scarring of various tissues and is attributed to excess deposition of ECM components including collagen [4]. Fibrosis is also an impartial characterization of the wound healing process that occurs due to the persistence of chronic injury for several months or can also result from other chronic diseases. The older concept of fibrosis focused on the production of ECM, but current studies point to epithelia as the culprit by producing new fibroblasts. Fibroblasts that are derived from the bone marrow can also arise due to the EMT of cells at injury sites. The secondary epithelium in mature tissue undergoes EMT and produces fibroblasts which ultimately leads to fibrogenesis. Considerable evidence exists that is mainly related to progressive kidney disease as well as chronic diseases of the liver and lung [26]. Although current treatments for fibrotic diseases such as idiopathic pulmonary fibrosis, liver cirrhosis, systemic sclerosis, progressive kidney disease, and cardiovascular fibrosis typically target the inflammatory response, there is accumulating evidence that the mechanisms driving fibrogenesis are distinct from those regulating inflammation. In fact, some studies have suggested that ongoing inflammation is needed to reverse established and progressive fibrosis [4]. A timely review was published on the "fibroblast conversion" concept (with an emphasis on renal fibrosis), in which epithelial cell transdifferentiation was shown to be important in the production of myofibroblasts, the major cells implicated in organ fibrosis. They are responsible for the natural accumulation of ECM in granulation tissue, as well as the excessive deposition of interstitial ECM in pathologic situations [27]. Myofibroblasts die during normal wound healing, and their apoptosis occurs in tandem with the epithelialization stage of the repair (Figure 3). Myofibroblasts, on the other hand, produce a collagen-rich stiff scar in the context of pathological scarring, which affects tissue architecture and changes the biochemical and biophysical milieu, resulting in dysfunctional tissue. Deregulated myofibroblast activity compromises tissue function and leads to organ failure in the long run [28].

Epithelial-mesenchymal transition and oral submucous fibrosis

Inflammatory cytokines generated in response to inflammation may have a role in OSMF progression via multiple EMT pathways [29]. The degenerative alterations in the connective tissue of OSMF have been described as having the potential to impact the overlying epithelium and lead to EMT [30]. Keratinocytes have been scrutinized as mere victims of oral mucosal injury, whereas fibroblasts have been classically observed as the main cells responsible for the structural and functional alterations of the oral mucosa [8]. Keratinocytes, in response to areca nut chewing, secrete some chemical mediators such as IL-6, prostaglandin E2 (PGE2), and TNF-α, which initiate inflammation of the mucosa [31]. They are also the main source of TGF-β1 which triggers both increased collagen production and decreased degradation pathways that promote ECM assembly and remodeling [32,33]. Several studies have investigated the abnormal expression of some cytoskeletal proteins, such as vimentin and cytokeratin, and some ECM molecules, such as tenascin, fibronectin, and MMP-2, MMP-9, and plasminogen activator inhibitor-1 (PAI-1) may also be associated with these morphological changes of keratinocytes. Many cytokines, nucleus proteins, and signaling pathways involved in EMT have been expressed and activated in OSMF proving the fact that EMT contributes to the pathogenesis of EMT [34]. These markers of EMT may prove to be efficient targets to control further progression and improve the prognosis of OSMF.

TGF-β is a pluripotent factor that can induce various biological responses in the same cell type. TGF-β may cause both cell death and EMT in epithelial cells. TGF-β communicates with downstream mediators called Smad proteins via a pair of transmembrane receptor serine-threonine kinases. Smad2 and Smad3 are receptor-activated Smad proteins that are phosphorylated directly by the TRI receptor kinase, interact with the common mediator, Smad4, and translocate to the nucleus, where they play a key role in activating TGF-dependent gene targets [34]. Many studies have demonstrated that TGF-β is upregulated in OSMF tissues [35-37]. The activation of TGF-β is expressed by the nuclear localization of p-Smad2 [38]. TGF-β signaling is induced by ANEs in epithelial cells, with higher levels of p-Smad2, indicating stimulation of TGF-β ligand (TGF-β2) and its activator thrombospondin1 (THBS-1), leading to TGF-β pathway activation. Thus, there is a pro-fibrotic cascade involving the TGF-β pathway triggered in the epithelium that influences the underlying submucosa for a fibrotic response [39,40].

In an in-vitro study model, stimulation using ripe ANE led to the activation of nuclear factor kappa B (NF-κB) in both the OECM-1 and SAS cell lines, while ERK, JNK, and late p38 activation were observed in only OECM-1. It was also seen that ROS can stimulate the production of TGF-β1 by activating MAPK and ERK-directed Smad2 which moderates EMT. Many studies have demonstrated that, through phosphorylation of p-AKT, p-ERK, and p-p38, areca nut and arecoline activate the ERK/JNK/p38 MAPK pathways, and activation of these pathways is important in OSMF and OSCC [41,42]. Oligonucleotide microarray was examined in OSMF where the expression profile of 14,500 genes was analyzed. The results showed that around >16 genes were upregulated and 149 genes were downregulated in OSMF. Several genes involved in EMT induced by TGF-β were identified including secreted frizzled-related protein-4 (SFRP4), thrombospondin 1 (THBS1), MMP-2, and Charcot Leyden Crystal-18 (CLC-18), suggesting its important role in the pathogenesis and malignant transformation of OSMF [19].

EMT has been implicated in the development of ECM-producing cells such as fibroblasts and myofibroblasts that lead to fibrosis in OSMF [43]. Arecoline causes increased Twist expression in buccal mucosal fibroblasts and may be responsible for the myofibroblastic phenotype via EMT, thus contributing to the pathogenesis of OSMF. EMT attributes in OSMF include reduced E-cadherin and β-catenin levels along with increased N-cadherin and Twist levels [44,45]. The Snail1 and Snail2 (Slug) are two transcription factors that belong to the snail superfamily, and consist of a highly conserved C-terminal domain with zinc fingers that bind to the E-box motif in the target gene promoters. However, in fibroblasts of the fibrotic buccal mucosa, the snail binds to the E-box in the alpha-SMA promoter and brings about upregulation of myofibroblast expression, thus perpetuating fibrosis [46]. E‑cadherin, Twist 1, and Snail 1 have been evaluated immunohistochemically in OSMF tissues, and their association with malignant transformation has been demonstrated, suggesting their role in EMT [47]. A previous study demonstrated an increase in the expression of Snail 1 and Twist with the concomitant loss of E-cadherin [48]. In addition to the direct effects of transcription factors on gene expression, there also occur some changes at the RNA level to regulate EMT progression such as alternative splicing of nascent RNAs into mRNAs that generate proteins with structural and functional differences in EMT. Moreover, miRNA-mediated degradation of gene transcripts defines the activities of key proteins in the control of EMT [49]. The downregulation of two RNA-binding proteins, ESRP-1 and ESRP-2, results in mesenchymal protein isoforms that help define alterations in adhesion, motility, and signaling pathways. miRNAs also target genes that help to define the epithelial or mesenchymal phenotype, such as those encoding adhesion junction and polarity complex proteins and signaling mediators.

Microarray investigation also showed upregulation of Twist, ZEB1, and MMP-9 along with downregulation of miRNA-205 in OSMF. Moreover, miR-203 inhibits arecoline-induced EMT. Significant downregulation of miR-203 was noticed in OSMF, suggesting its role in the pathogenesis of EMT [50,51].

Therefore, in OSMF, EMT denotes the phenotypic conversion of epithelial cells into myofibroblast-like cells after the loss and gain of certain molecular markers due to chronic cell injury caused by ANE which produces aberrant amounts of ROS and, in turn, triggers both MAPK and NF-κB pathways.

Epithelial-mesenchymal transition and oral squamous cell carcinoma

The role of EMT in head and neck squamous cell carcinoma (HNSCC) has been studied and evaluated in the past. E-cadherin expression was strong and membranous in well and moderately differentiated OSCC and broad invasive fronts, whereas the staining was less intense with both membranous and cytoplasmic localization in poorly differentiated OSCC, and weak or negative in sarcomatoid carcinomas, as well as in invasive fronts showing finger-like and individual tumor cell proliferation. Vimentin was negative to mild in well and moderately differentiated tumors and in broad-based and smooth invasive fronts, while in poorly differentiated OSCC, increased cytoplasmic vimentin stain was seen, as well as in finger-like invasive fronts. Sarcomatoid carcinomas had the lowest E-cadherin and intense vimentin expression. A significant correlation was detected between vimentin expression and poor histologic differentiation, finger-like invasive fronts, as well as marginally with lymph node involvement and stage of the tumor. The above expression was associated with Src activation, suggesting that its activation results in the downregulation of E-cadherin and upregulation of vimentin leading to EMT. The study concluded that these changes can occur not only in pre-invasive dysplastic lesions but also persist and contribute to the development of aggressive and invasive phenotypes [52].

Double immunostaining with epithelial membrane antigen (EMA) and α-SMA in tongue OSCC was also studied for EMT. There were 41% of cases that exhibited cells that co-expressed both the markers and presented with mesenchymal morphology, suggestive of cells that have undergone EMT. These cells were associated with abundant stromal myofibroblasts around them. Further, enhanced TGF-β expression was also observed along with the co-expressing epithelial cells and stromal myofibroblasts, suggesting its role in the emergence of both these phenotypes [53].

In a study, it was seen that the markers of EMT are common in both primary as well as metastatic tongue OSCC. They found upregulation of cadherin 11 (mesenchymal cadherin) and FSP-1 along with downregulation of Syndecan I, resulting in decreased intercellular adherence and increased acquisition of motility and invasion. Twist being a transcriptional factor represses E-cadherin expression and secreted protein acidic cysteine-rich osteonectin (SPARC), a matricellular protein that downregulates E-cadherin with the enhancement of MMP expression in primary and metastatic tongue OSCC. Along with EMT, carcinoma-associated fibroblasts (CAF) were also found that act as co-migrators producing a pathway for invasion and metastasis. Cadherin 11 and FSP-1 were also upregulated in the tumor microenvironment and were expressed by fibroblasts, endothelial cells, inflammatory cells, and ECM, suggesting a synergistic relationship between carcinoma and the microenvironment in EMT. Zhou et al. showed that CAFs induced the upregulation of EMT markers such as fibronectin and vimentin and the downregulation of E-cadherin in tongue SCC cell lines. Ding et al. suggested that α-SMA positive myofibroblasts have a role in the progression, metastasis, and survival of tongue OSCC by promoting EMT. A similar finding was reported by Li et al. who suggested that CAFs can promote EMT in tongue OSCC. Chaw et al. demonstrated that EMT markers, such as E-cadherin, β catenin, APC, and vimentin, are involved in oral carcinogenesis via Wnt pathway dysregulation. Reduced membranous and increased cytoplasmic/nuclear expression of β-catenin, reduced E-cadherin expression, aberrant expression of APC, and enhanced vimentin expression were evidenced from normal to potentially malignant to malignant lesions, suggesting a role of EMT in carcinoma progression [54-56].

A significant correlation was observed between the cadherin switch and histologic differentiation, invasion patterns, and the presence of lymph node metastasis in HNSCC [57]. Reduced E-cadherin and its cytoplasmic expression associated with histologic differentiation and the advancing edge of the tumor are inversely correlated with vimentin reactivity which is increased with tumor differentiation and invasive fronts. Low levels of N-cadherin expression were evidenced more in poorly differentiated tumors and at the advancing edge predominantly in the cytoplasm which was contradictory to that of E-cadherin, suggestive of cadherin switch. These findings suggest that these markers signify EMT and can be used for prognostic stratification of patients with tongue OSCC [58]. This was in contrast with a study that showed that, although there was a reduction in E-cadherin expression at the invasive front which was associated with poor differentiation, N-cadherin positivity was minimal and showed no correlation with the clinicopathologic parameters. They suggested that reduced E-cadherin cell-cell adhesion is an important determinant of oral cancer progression but not cadherin switch [59]. The progressive reduction of E-cadherin with a grade of OSCC, i.e., well-differentiated SCC (83.3%), mildly differentiated SCC (61.9%), and poorly differentiated SCC (33.3%) which was associated with increased vimentin reactivity with differentiation, i.e., well-differentiated SCC (25%), mildly differentiated SCC (33.3%), and poorly differentiated SCC (66.7%), was associated with lymph node metastasis. Further, E-cadherin positivity was more in the center of the tumor compared to the infiltrative margin, whereas vimentin expression was higher in the infiltrative margin compared to the central areas (p < 0.05). Thus, there was a negative correlation between E-cadherin and vimentin in OSCC, suggestive of EMT [60].

The prognostic value of three markers associated with EMT phenotype, i.e., E-cadherin, β-catenin, and EGFR was also assessed. The study found that high E-cadherin expression is an independent predictor of overall survival in HNSCC. A significant correlation was also observed between E-cadherin and β-catenin expression, and a positive correlation was observed between E-cadherin and EGFR expression. E-cadherin and β-catenin interaction affects the cell-cell adhesion and activates the WNT signaling, whereas EGFR/E-cadherin/catenin complex seems to regulate the MAPK activity, thus playing a role in cancer progression via activation of EMT [61].

The expression of E-cadherin, N-cadherin, and vimentin in the invasive front and superficial central areas of OSCC was also studied. A reduced E-cadherin expression was noted in 75% of the cases which was more prominent in the invasive fronts and in tumors with high invasiveness. N-cadherin was negative in all samples, while vimentin positivity was reported in 30% of OSCC cases, but no difference was noted between invasive fronts and central areas, as well as between high and low invasive tumors. No correlation was found between the markers at the invasiveness front as well as with the tumor stage and lymph node status. The study suggested that reduced E-cadherin is a noteworthy EMT marker in OSCC [62].

EMT in multiple primary OSCCs has also been evaluated using immunohistochemical markers E-cadherin, catenin, APC, collagen IV, Ki-67, cyclin D1, and CD44 by tissue microarray. Significant associations were observed among multiple OSCC and E-cadherin, β-catenin, APC, and cyclin D1 expressions. There was significantly lower survival for patients with multiple OSCCs for tumors showing negative expression for E-cadherin and β-catenin. Multivariate survival analysis revealed prognostic interdependence of E-cadherin and β-catenin downregulation in predicting worst overall survival. A correlation between tumor satellites and EMT in tongue cancer was also demonstrated which suggested that loss of epithelial characteristics contributes to tumor satellite formation and acquisition of EMT allows its spread and invasion. Similarly, in a study, the presence of tumor budding in 71.7% of tongue OSCC cases was associated with clinical stage, size of the tumor, histologic differentiation, as well as the presence of regional lymph node metastases, and reduced survival rates. Further, in areas of tumor budding, significant deregulation of E-cadherin and vimentin expressions was also noted. The study concluded that tumor budding representing EMT is an adverse prognostic feature for tongue OSCC [63].

Mechanism of epithelial-mesenchymal transition in oral squamous cell carcinoma

Overexpression of Snail in OSCC cell lines causes loss of E-cadherin expression, fibroblastic morphology, and upregulation of vimentin, indicating EMT induced by Snail. Additionally, the cells show higher levels of MMP-2 leading to increased invasive capacity. Thus, Snail acts on cell-cell adhesion as well as invasion capacity via EMT [64]. Snail alone can accomplish a complete EMT, i.e., Snail-transfected cells showed fibroblastoid appearance, vimentin filaments and E-cadherin/N-cadherin switch, lack of hemidesmosomes, and upregulation of ZEB-1 and ZEB-2, which are E-cadherin repressors [65]. Snail-induced EMT causes downregulation of the laminin-5 chain and laminin- 4 chains. This laminin-4 inhibited the cell adhesion to other ECM proteins such as laminin-5 and fibronectin, thereby leading to invasive behavior [66]. Snail expression in the stroma of OSCC was associated with the development of myofibroblast phenotype by EMT [67]. TGF-β1-triggered EMT in OSCC cells is regulated by Snail and Slug. Snail may upregulate MMP-2 or MMP-9 initiating EMT, whereas Slug synchronizes with Snail for maintaining EMT for a longer time via stimulation of MMP-9 activity [68]. Integrin expression can induce EMT in OSCC characterized by the acquisition of fibroblast-like morphology, increased expression of vimentin, and reduced expression of epithelial markers such as keratin and E-cadherin [69].

EGF and TGF-β co-stimulation can cause EMT in OSCC cells. The phenotype transition shows reduced E- cadherin levels and enhanced vimentin expression at the protein level along with cell scattering. Additionally, the cells displayed enhanced invasive capacity and adhesion to type I-IV collagens. Enhanced expression of laminin 332γ2 was also seen in these cells [70].

Integrin-linked kinase (ILK) plays a key role in integrin-mediated cell-ECM interaction regulating various functions. ILK has been implicated in EMT by upregulation of Snail which, in turn, causes altered E-cadherin as well as N-cadherin expression [71].

Overexpression of membrane type-1 MMP (MT-1 MMP) can induce EMT in SCC-9 cell lines characterized by increased Twist and ZEB that bring about the repression of E-cadherin leading to low adhesive and invasive capabilities. Further, these cells showed cancer stem cell-like characteristics, such as low proliferation, self-renewal, chemotherapeutic resistance, resistance to apoptosis, etc. [72]. MiRNAs have an important role in EMT. MiR-200 family members suppress EMT by targeting ZEB1 and ZEB2. MiR-300 is downregulated in EMT, with its expression inversely correlated with Twist. Reduced expression of MiR-639 was seen in TGF-β-related EMT [73].

Oct-4 is a key transcription factor involved in the maintenance of pluripotency and self-renewal in undifferentiated embryonic stem cells. It is over-expressed in diverse cancers and is an important determinant of cancer stem cell properties. Oct-4 can induce EMT leading to repression of E-cadherin and enhanced N-cadherin and Slug expression [74].

Recent advances in science and research have heightened our understanding of the fundamentals of mechanisms involved in chronic injury and fibrosis in the oral mucosa. Understanding at the molecular level will help in the therapeutic control of EMT to promote tissue regeneration, treat fibrosis, and prevent cancer metastasis [75]. An innumerable number of tumor EMT inhibitors have been found in in-vitro studies, many of which are difficult to apply clinically because of stability and targeting issues [76].

## Conclusions

EMT reversal is based on the idea of removing mesenchymal cells that have developed treatment resistance and cancer stem-cell-like features. One practical and philosophical consideration in developing an EMT readout assay is how much and how far EMT should be reversed. Robust quantitative measurements of intended endpoint readouts with high reproducibility and tight sample variability are critical for assay development success. Any EMT drug discovery assay would have to take these factors into account.

EMT is an essential step in both physiological and pathological events. Pathological EMT can be regarded as the reactivation of developmental programs in adults. Surprisingly, the same pathways that help in tissue regeneration can be a nightmare if the injury persists for a longer time. One of the critical areas of research is to understand complex molecular events associated with EMT. This requires an interdisciplinary approach in which various cellular and biological methods are combined with emerging technologies. Fibrosis is an important event that is a consequence of chronic inflammation and has different debilitating outcomes. Removal of the causative agent that is causing chronic inflammation is the first and most important step in the treatment of OSMF and the prevention of its malignant transformation. Second, the role of EMT in the pathogenesis of OSMF cannot be ignored. Understanding the pathways can halt the EMT events at an early stage or reverse the process of EMT. Both the removal of chronic causative agents and the halting of EMT pathways can help in the prevention of the progression of OSMF to malignancy. EMT research in the next few years can be encouraging as new mouse models and molecular probes are identified to address the unanswered questions.
